# Genomics of human longevity

**DOI:** 10.1098/rstb.2010.0284

**Published:** 2011-01-12

**Authors:** P. E. Slagboom, M. Beekman, W. M. Passtoors, J. Deelen, A. A. M. Vaarhorst, J. M. Boer, E. B. van den Akker, D. van Heemst, A. J. M. de Craen, A. B. Maier, M. Rozing, S. P. Mooijaart, B. T. Heijmans, R. G. J. Westendorp

**Affiliations:** 1 Section of Molecular Epidemiology, Leiden University Medical Center, Leiden, The Netherlands; 2 Center for Human and Clinical Genetics, Leiden University Medical Center, Leiden, The Netherlands; 3 Department of Gerontology and Geriatrics, Leiden University Medical Center, Leiden, The Netherlands; 4 Netherlands Consortium for Healthy Ageing, PO Box 9600, 2300 RC Leiden, The Netherlands

**Keywords:** human longevity, longevity genomics, epigenetics and ageing

## Abstract

In animal models, single-gene mutations in genes involved in insulin/IGF and target of rapamycin signalling pathways extend lifespan to a considerable extent. The genetic, genomic and epigenetic influences on human longevity are expected to be much more complex. Strikingly however, beneficial metabolic and cellular features of long-lived families resemble those in animals for whom the lifespan is extended by applying genetic manipulation and, especially, dietary restriction. Candidate gene studies in humans support the notion that human orthologues from longevity genes identified in lower species do contribute to longevity but that the influence of the genetic variants involved is small. Here we discuss how an integration of novel study designs, labour-intensive biobanking, deep phenotyping and genomic research may provide insights into the mechanisms that drive human longevity and healthy ageing, beyond the associations usually provided by molecular and genetic epidemiology. Although prospective studies of humans from the cradle to the grave have never been performed, it is feasible to extract life histories from different cohorts jointly covering the molecular changes that occur with age from early development all the way up to the age at death. By the integration of research in different study cohorts, and with research in animal models, biological research into human longevity is thus making considerable progress.

## 1. Introduction

Genomic studies into human longevity are inspired by the fact that, in animal models, healthy lifespan has proved to be remarkably plastic, and major pathways of lifespan regulation have been identified. Considerable lifespan extension has been induced in models as diverse as yeast, worms, fish, flies and rodents by applying genetic manipulation and dietary restriction (DR) (see [1] for review). Reduced activity of nutrient-sensing pathways such as insulin/insulin-like growth factor (IGF-1) signalling (IIS) and target of rapamycin (TOR) signalling mediated lifespan extension, and also the extension of lifespan by DR [2]. An interesting observation from the perspective of human ageing is that, in rodents and monkeys, diets restricted in glucose, fat or protein uptake reduced or delayed the risk of cancer and metabolic disease, thus extending the healthspan of the animals [2]. Following the discovery of genes and pathways involved in animal lifespan extension, human research has focused on the corresponding candidate human genes with genetic, genomic and epigenetic studies into ageing and longevity. The designs of these studies differ with respect to the selection of naturally occurring phenotypes and the study populations, which include population-based, patient-based, family-based and exposure-based cohorts.

Studies into human age-related disease phenotypes are focused on population-based and patient-based cohorts of elderly individuals in order to find mechanisms that contribute to the onset and/or progression of complex common diseases. For all common diseases, calendar age is a major risk factor and many disease-related parameters, such as serum glucose level, typically change as a function of age. Attempts are being made to interpret which combination of age-related parameters reflects the biological ageing rate of individuals and which of these biomarkers of ageing could influence the onset of disease and of co-morbidity. Such molecular epidemiology studies are combined with genomic approaches to identify the biological mechanisms that may be causally involved in the onset of morbidity and mortality. These investigations require large study cohorts that allow stratifications for age and sex with repeated confirmation of observations in independent studies and, as we shall discuss here, cover research into life-history changes from conception until death.

Developmental processes and early-life programming may contribute to the onset of disease later in life. Epidemiological studies have associated characteristics of early development such as birth weight with the occurrence of later disease [3]. Another example originates from genetic research into the aetiology of osteoarthritis, a common joint disease. A number of the genes identified for this disease are active in the growth plate and silenced in adult life. Disease susceptibility variants are expected to contribute early in life to mild skeletal malformations and later in life to affect homeostasis of articular cartilage [4].

In addition to the late effects of developmental processes, ageing is likely to be affected by longevity assurance mechanisms. In the last decade, human longevity studies have been initiated in cohorts of individuals and their family members to find protective mechanisms delaying the ageing-related phenotypes and disease onset in highly and exceptionally aged subjects.

## 2. Designs to study parameters of healthy ageing, morbidity, mortality and longevity

Human cohorts may vary considerably in their morbidity, mortality and longevity characteristics and yet they have shown a common increase in mean life expectancy in the past two centuries [5]. This is mainly due to improved hygiene, nutrition and healthcare. There is a large variation in healthy lifespan among the elderly and remarkably exceptional longevity (EL) can be reached with a low degree of age-related disability [6,7]. Heritability studies comparing the concordance of lifespan in monozygous and dizygous twins estimated a 25–30% genetic contribution to human lifespan variation [8–11], which becomes increasingly important at higher ages. The most prominent genetic influence is present in families in which survival to high ages clusters [12,13]. Unlike model systems where single-gene mutations have major life extension effects, human longevity is presumed to be a complex trait [14].

The design of studies aimed at the extraction of molecular parameters determining biological ageing and longevity includes the cross-sectional designs comparing unrelated humans in groups of different calendar age. Such studies may also include follow-up data that must be considered the more unbiased design to associate biological parameters with morbidity and mortality (Leiden 85 plus Study, Danish 1905 cohort). The cross-sectional design is especially sensitive to differences in cohorts confounding the associations one is aiming to observe. Controls from the birth cohort of long-lived individuals are now deceased, so younger controls originate by definition from other birth cohorts than the long-lived individuals. As a result of high mortality and changes in the mortality rate at higher ages, a true observation of how a parameter associates with survival in a population can be obtained only by a combination of prospective studies with increasing baseline ages.

To improve the design for genetic studies, families enriched for familial longevity are being investigated. These have been selected by either recruiting nona/centenarians and their middle-aged offspring (the Ashkenazi Jewish Centenarian Study) or nonagenarian sibling pairs and their middle-aged offspring (Leiden Longevity Study, Genetics of Healthy Ageing (GEHA)/Mark-Age study). The offspring of long-lived individuals representing (as a group) cases predisposed to longevity can now be compared with middle-aged controls (spouses) from the same geographical background and birth cohort to investigate which age-related phenotypes actually associate with familial longevity. Resembling the ability of a time machine to observe what happened to a person years ago, this study design allows the multi-generational analysis of molecular and clinical parameters specific for long-lived individuals and their family members at elderly, middle and younger ages.

**Figure 1.**
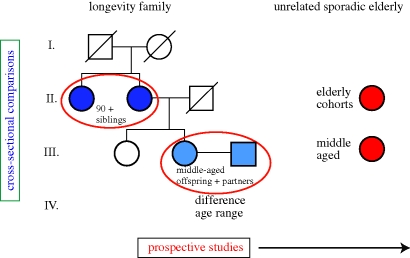
Study design of the Leiden Longevity Study: from LLS phenotypes to biomarkers.

One of the studies on which we focus in this paper is the Caucasian family-based Leiden Longevity Study (LLS; figure 1) in which long-lived siblings were recruited together with their offspring and the partners thereof. Families were recruited if at least two long-lived siblings were alive and fulfilled the age criterion of 89 years or older for males and 91 year or older for females, representing less than 0.5 per cent of the Dutch population in 2001. In total, 944 long-lived proband siblings were included with a mean age of 93 (89–103), 1671 offspring of a mean age of 59 (34–80) and 744 partners of a mean age of 59 (30–79).

The study design (figure 1) shows that, in cross-sectional studies, phenotypes can be compared between familial nonagenarians and sporadic elderly from the population (such as from the Leiden 85 plus Study) to distinguish familial longevity markers from parameters marking all long-lived individuals. Parameters can also be compared between offspring and partners (as controls sharing a significant portion of socio-economic environment) or, to avoid missing associations owing to assortative mating, between offspring and other middle-aged controls from the general population. The association analyses can also be performed in the context of the survival history of the family; in which case a longevity phenotype is most prominent in families expressing the most extended survival (by a combination of exceptional ages at death and the number of family members reaching such high ages). Convincing evidence for a phenotype to be a marker of biological age is provided if prospective studies show that baseline variation in the phenotype associates with morbidity or mortality in the time to follow-up (figure 1).

## 3. Phenotypes reflecting (un)healthy ageing and familial longevity at middle age

The exceptional ageing process in long-lived individuals and their family members is expressed both at high and middle age. The survival benefit of the families from the Leiden Longevity Study (LLS) is marked by a 30 per cent decreased mortality risk observed in the survival analysis of three generations, i.e. the parents of the sibling pairs, their deceased additional siblings and their offspring [13]. The familial survival advantage thereby exceeds the increased lifespan expectancy in the last generations of western societies due to non-genetic factors. The offspring of the long-lived siblings further have a lower prevalence of diabetes, myocardial infarction, hypertension and also use less medication for cardiovascular disease as compared with their partners, with whom they have shared decades of common environment [15]. Others have observed the same for offspring of centenarians who in addition showed also a low prevalence of cancer [16,17].

We will now discuss the comparison of parameters in offspring of long-lived individuals and their partners, which may indicate the pathways contributing to familial longevity. Since a biobank was set up for the LLS study, parameters can be established in DNA and RNA isolated from whole blood, serum, urine, peripheral blood mononuclear cells (PBMCs) and primary fibroblast strains from skin biopsies. In addition to more classical parameters, e.g. parameters of glucose and lipid metabolism, the ongoing phenotyping includes lifestyle questionnaires, photographs, imaging (magnetic resonance imaging of the brain, X-ray of joints), metabonomics (NMR- and mass-spec-based) and proteomics.

## 4. IIS signalling parameters in longevity families

On the basis of the observations on lifespan extension in animal models, the most likely favourable biological features expected to associate with human longevity would be parameters in IIS signalling and growth, thyroid metabolism and cellular stress resistance. Indeed various molecular and cellular phenotypes were found to mark the altered and probably more healthy ageing process expressed in the longevity families. Phenotypes in offspring that change as a function of age showed patterns usually represented by younger subjects, as if ageing occurs at a slower rate. The non-diabetic middle-aged offspring had a lower prevalence of metabolic syndrome, lower mean fasting blood glucose and insulin levels, a higher mean insulin sensitivity and a more favourable glucose tolerance than their partners. The two groups did not differ with respect to age, sex distribution, body mass index (BMI) and lifestyle indices such as the level of physical activity and smoking behaviour [18,19]. No differences in serum IGF-1 levels or height were observed between the total group of offspring and controls. This observation is in contrast with a previous Ashkenazi Jewish Centenarian Study from which it was concluded that owing to reduced IGF-1R signalling, IGF-1 levels were increased in female offspring of centenarians with a concomitant lower height [20], potentially explained by rare genetic variants in the *IGF-1R* gene.

## 5. Lipid and thyroid metabolism in longevity families

Parameters of lipid metabolism (i.e. classical serum lipids, lipoprotein particle sizes and ApoE levels) have been associated with calendar age, cardiovascular health and longevity in human studies. From these studies, it became clear that serum apolipoprotein/lipid-based cardiovascular risk factors are different (and even reversed) for middle-aged and elderly cohorts.

We and others measured lipoprotein particle size and analysed the relationship with longevity [21,22]. Recently, we have completed multivariate analyses of these parameters in the total LLS study population accounting for correlations with classical lipid parameters. Offspring had larger low-density lipoprotein (LDL) particle sizes and lower triglyceride levels, indicating that they carry a more beneficial lipid profile. For comparison, individuals suffering from metabolic syndrome have the opposite profile. In the multivariate analysis, we found that, among all lipid parameters, LDL particle sizes particularly associated with familial longevity in males, whereas low triglyceride levels marked female longevity. Thus, lipid metabolism plays a role in human familial longevity.

The altered glucose and lipid metabolism of longevity families and the preservation of insulin sensitivity with increasing age resemble the phenotypes of long-lived dietarily restricted rodents and primates (see [2] for review). This would predict that not only nutrient-sensing pathways such as IIS but also TOR signalling may be involved in human longevity. Evidence to support this hypothesis is emerging from our genomic studies in the LLS cohort as discussed below. In addition, our observations may imply that the hypothalamo-pituitary-thyroid axis is involved in these features. Indeed, lifespan extension in animal models associates with low activity of the thyroid hormone axis and in our studies we observed that the middle-aged offspring have lower serum-free triiodothyronine levels compared with their middle-aged partners, suggesting that a low thyroid hormone metabolism may contribute to familial longevity at an early age [23].

A better understanding of pathways that contribute at middle age to the divergence of healthy and unhealthy ageing humans may be substantiated by in depth studies of the cells and tissues of longevity family members in the context of their genomic background.

## 6. Genetic studies of human longevity

Genetic and genomic studies into longevity have been performed based on a hypothesis, referred to as a candidate gene approach. Alternatively, explorative genome-wide analyses have been applied in which genetic variation and gene transcription across the complete genome are being studied for associations with longevity and related traits. Genetic studies into human disease and longevity include candidate gene approaches, genome-wide association studies (GWASs) and genome-wide linkage studies.

Such studies in long-lived individuals and their families may provide more insight into the pathways that drive the combination of seemingly beneficial physiological parameters. Longevity may be explained by the presence, in the genetic make-up of LLS family members, of alleles protecting against age-related phenotypes and diseases that contribute to population mortality. An alternative explanation may be simply the absence of alleles promoting such diseases. Now that the first wave of GWASs have generated a catalogue of common genetic variants (single nucleotide polymorphisms, SNPs) that associate with the major diseases that contribute to human mortality, it can be tested whether the disease susceptibility alleles are absent from the genome of long-lived individuals. We investigated in nonagenarians from the family-based Leiden Longevity Study and octogenarians from the population-based Leiden 85 plus Study the frequency of a set of alleles currently known to increase the risk of coronary artery disease, cancer and type-2 diabetes as identified by GWASs. Both the nonagenarian siblings from long-lived families and the sporadic long-lived individuals appeared to carry the same number of disease risk alleles as young controls, indicating that longevity was not easily explained by a remarkable absence of disease susceptibility alleles. More likely, therefore, the genome of the long-lived harbours longevity-promoting alleles. Candidate gene and genome-wide approaches have been applied to identify such alleles. The frequency of genetic variants was typically compared between highly aged cases and young controls, revealing loci at which genetic variants may contribute to a higher or lower probability of survival into old age. So far, this approach has mainly been applied to study single candidate genes such as the mammalian orthologues of loci in IIS signalling pathways that emerged from lifespan extension studies in animal models. An interesting observation that needs to be taken into human studies is the fact that those protective ‘longevity’ genes in animals indeed exist but do not affect mortality at all ages [24], not equally in both sexes and associate often with reduced body size.

The effect of reduced IIS signalling on lifespan extension in model systems is through changes in gene expression and especially genes orthologous to human *FOXO* transcription factor, *HSF-1*, a heat shock transcription factor, and *NFE2L2* [25], a xenobiotic response factor. The initial human candidate longevity gene studies were dominated by contradictory results [26]. The more consistent evidence obtained by repeated observation in independent cohort studies for association to longevity was found for the *APOE* locus and, more recently, the *FOXO1* and *3* [27–29] and *AKT1* loci [30]. The effect size of the association of the *FOXO3* variant appears to vary with the age of the cases, being most prominent in centenarians. Other intriguing observations that need to be replicated but fit observations in humans at the phenotype level discussed above were made in the Ashkenazi Jewish Centenarian Study in which a higher serum thyroid-stimulating hormone level and *TSHR* genetic variation marked the centenarian population [31]. Recently, an association with longevity was found for genetic variation in RNA-editing genes [32].

A more complete understanding of the relevance of genetic variation in influencing population-wide mortality and/or population-wide EL may come closer when cohort studies can be joined in an analysis of the combined effects of multiple SNPs at age strata covering the whole lifespan and stratified by sex.

Previously, a hypothesis-free genome-wide linkage scan reported evidence for linkage to longevity on chromosome 4q25 [33]. These results, obtained in extended longevity families, could not be replicated in nonagenarian sibling pairs [34] and have to be further investigated in larger longevity families. More linkage studies are under way in the GEHA population [35] and the LLS study.

A number of GWASs applying state-of-the-art GWAS protocols are also under way. A standard procedure has been set for GWAS studies in which accepted levels of statistical significance and credible top hits can only be obtained by a meta-analysis of several large cohort studies, to avoid reports on false positive associations caused by multiple testing and genotyping errors that occur as a consequence of high-throughput technology. A GWAS meta-analysis of four GWASs of survival to age 90 years or older has been performed, but has not yet reported on genome-wide significant associations [36]. A more recent GWAS was performed on a study of 1055 centenarian cases and 1267 controls [37]. Within this dataset, consisting of a learning set and a small testing set, a genetic model was generated by which 150 SNPs described EL and predicted EL in the test set. Since this study is underpowered and the results were not confirmed by replication in independent cohorts following standard procedures for GWAS studies, it is as yet highly unlikely that the genetic signature predicts longevity in other studies and that the top hits represent true new leads for longevity research.

More GWA data are being collected for longevity. Joint analyses of these studies will be necessary to disentangle this complex trait and to allow for a testing of the joint associations of genes in a pathway, which is a more appropriate analysis for a complex trait than investigating single genes.

## 7. Genomic studies of human ageing and longevity

Genome-wide expression profiles have been studied in brain, lymphocytes, kidney and skeletal muscle tissues from individuals of different ages [38–42]. Since the expression of some genes reflected the functionality of the specific organs they originated from, such genes may not only mark the chronological ages of subjects but also their tissues' biological age. Although all studies reported gene expression changes to occur as a function of age, the comparison of offspring of long-lived subjects and their partners may provide a step further towards the identification of early and possibly causal contributions to the ageing process [43].

**Figure 2.**
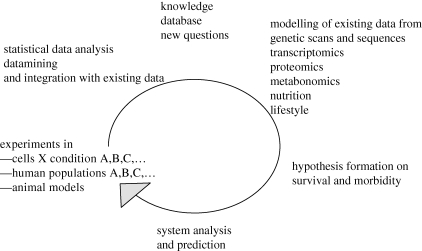
Linking molecular epidemiology to cell biology.

Gene expression profiles in RNA from whole blood samples were compared between 50 nonagenarians and 50 controls from the LLS study to investigate differential gene expression that may arise as a function of age. Differential expression was observed for about 7 per cent of the analysed probes. In our unique study design, we also tested which of these age-related changes is an early marker of longevity by comparing gene expression profiles of 50 offspring of the nonagenarians with the controls. Gene expression differences could be observed in offspring and partners, especially in interaction with age. Among the limited number of differentially expressed genes, we observed decreased expression of genes in the mTOR pathway in the members of long-lived families. These results, when confirmed, would suggest a role for TOR signalling in human ageing and lifespan regulation possibly in ways comparable to its role in lifespan regulation in other species. This would be supported by the observation that LLS family members express DR-like phenotypes (such as preserved insulin sensitivity). The initial genomic results originating from micro-array analysis of restricted sample sizes can now be followed (i) by testing for differential gene expression by RT–PCR in a much larger sample of offspring and controls (2500 in total), (ii) by testing for genetic association of *TOR* genes with longevity and longevity-related phenotypes in the LLS offspring generation (since GWAS data are available in all subjects of the study), and (iii) by investigating the pathway in primary fibroblasts of subjects from the LLS study. By investigating TOR and IIS signalling in fibroblasts of LLS subjects, functional alterations in specific proteins may be identified which can be tested in the genomic studies of the LLS cohorts, but also in other cohorts to establish a relation with human health. In this respect, we are combining the molecular epidemiological studies in human longevity with cell biological approaches (figure 2). Especially when performing such analyses in the context of the whole pathway, this approach may connect to systems biology approaches focused on the role of the TOR pathway in cancer, diabetes and ageing.

We reported that fibroblasts from the skin of LLS study subjects showed increased resistance to cellular stress, another feature of dietary and genetically induced lifespan extension in animal models. We investigated fibroblasts isolated from skin biopsies obtained from different donor groups: 20 and 90 year olds as well as middle-aged offspring and partners of the LLS [44]. Fibroblasts were exposed to rotenone and hyperglycaemia and assessed for senescence-associated beta-galactosidase (SA-beta-gal) activity, apoptosis/cell death and growth potential. Stress-induced increase in SA-beta-gal activity was lower in fibroblasts from offspring than from partners and stress-induced apoptosis/cell death was higher in fibroblasts obtained from offspring. The observations show that *in vitro* cellular responses to stress reflect the propensity for human longevity of the donor. In much the same way, we investigate whether this propensity is reflected in the TOR and insulin pathways and cellular phenotypes such as autophagy.

## 8. The influence of environmental factors and early development on human ageing and longevity

Genetic studies in humans have thus far not explained much of the considerable genetic component that was demonstrated for many physiological health conditions. Our insights may be improved by the study of rare variants in relevant genes, which has become feasible by applying novel and affordable deep and whole-genome sequencing technologies. To fully understand a complex trait such as longevity showing moderate heritability, it is also essential that the gene by environmental interactions be studied. Most of the human genetic studies have not yet focused on the analysis of such interactions (which requires large study samples), and this also requires more knowledge on the epigenetic regulation of the genome. Epigenetic marks on the genome regulate chromatin structure and accessibility of the DNA to the machinery regulating gene expression. This ‘epigenome’ can serve as a molecular archive of ongoing and past environmental conditions. Empirical data to link changes in the epigenome to disease and ageing are however still scarce. The best-characterized epigenetic marks are the methylation of cytosines in cytosine–guanine (CpG) dinucleotides and the modification of histones that package the DNA. These marks are heritable during cell division, particularly mitosis, and are involved in processes that require a stable control of gene expression, such as the selective gene-silencing during cell differentiation, parent-of-origin-specific silencing (imprinting) and suppressing the transposition of mobile elements (DNA sequences with the ability to copy themselves throughout the genome).

In keeping with early dedifferentiation theories such as that proposed by Richard Cutler, increases and decreases in DNA methylation occur as a function of age [45,46] and could mark a loss of epigenetic control and homeostasis [47]. Monozygous twins showed at ages between 3 and 74 years a divergence of genome-wide epigenetic and gene expression changes [48]. Such observations become especially relevant when they can be linked to the dysregulation of specific genes and to phenotypic consequences such as discordance for metabolic parameters. The study of one specific candidate gene for which the relevant DNA methylation sites are known revealed that variation in DNA methylation in human populations is heritable and changed only moderately as a function of age before 65 years [49]. This study focused on the well-characterized differentially methylated region of the imprinted insulin-like growth factor 2 (*IGF2*) gene, encoding a key factor in human growth and development. Following our observation concerning the *IGF2* gene, heritability of DNA methylation has also been shown in other studies. It will be difficult to prove how age changes in the epigenome contribute to human disease if the changes are frequent, stochastic and age-, tissue- or even cell-specific.

Numerous epidemiological studies have shown a link between characteristics of early development and the occurrence of disease later in life [3]. These observations form the basis for the Developmental Origins Hypothesis of Health and Disease (DOHaD), stating that adverse environmental conditions during specific windows of mammalian development can have lasting effects on metabolic pathways and physiology, thereby influencing the susceptibility to disease [50]. By studying specific methylation sites in a set of candidate genes involved in metabolism, we have shown in adult humans that the epigenome harbours molecular marks of past environmental conditions. We examined individuals exposed to the Dutch Famine during gestation in the Hunger Winter Families Study. At the end of World War II, a severe famine affected the western part of The Netherlands from November 1944 to May 1945. The average daily rations, which the authorities distributed during the famine, were less than 700 kcal (cf. normal daily requirements for women and men are 2000 and 2500 kcal, respectively). Prenatal exposure to the famine is associated with various adverse metabolic and mental phenotypes later in life, including a higher BMI, elevated plasma lipids, increased risks of cardiovascular disease and schizophrenia. Many of these associations depended on the sex of the exposed individual and the timing of the exposure during gestation. The first candidate locus investigated was again the *IGF2* gene. Individuals who were exposed to famine during early gestation had a lower *IGF2* methylation than controls six decades after the exposure [51]. Also, methylation at other loci was associated with prenatal exposure to famine [52]. Controls in these studies were same-sex siblings, thus minimizing confounding by sex, early family environment and the influence of genetic variation on DNA methylation. For the *IGF2* locus, we observed small increases in DNA methylation to be associated with maternal use of folic acid during periconception [53].

Determinants of developmental set-points that regulate epigenetic and homeostatic control may be common denominators for disease, healthy old age and longevity. The biological effects of variations in environmental conditions (such as nutrition, antigen exposure, parental age and lifestyle) should ideally be investigated systematically across the complete lifespan. Different human cohort studies cover the different relevant stages in life at which a candidate pathway can be investigated. The relevant stages that may influence morbidity and mortality later in life include both the prenatal and postnatal development (early-life programming) and the adult/ageing phase, determined by the combined consequences of events occurring during developmental stages and age-related changes contributing to a decrease in physiological capacity preceding diagnosed disease. The epigenetic and genomic changes that may have occurred in these phases would increase the variation between subjects leading to differences in morbidity, co-morbidity, mortality and longevity. It may be worthwhile to focus epigenetic and genomic studies in different life phases on longevity genes in IIS and TOR signalling, nutrient-sensing pathways that respond to environmental conditions. Possibly, this level of integrated research will provide an understanding of the preserved insulin sensitivity and DR-like phenotypes observed in long-lived individuals and their families and may reveal how to influence the underlying mechanisms for the benefit of elderly individuals in the general population.
